# Acute Salmonella typhimurium Aneurysmal Aortitis With Bacteremia, Pneumonia, and Thoracic Aortic Dissection in an Elderly Male

**DOI:** 10.7759/cureus.21431

**Published:** 2022-01-19

**Authors:** Sachin Patil, Maggie Wieser, Cliff Chen, Taylor B Nelson, Zach Holliday, William Roland

**Affiliations:** 1 Infectious Diseases and Critical Care, University of Missouri, Columbia, USA; 2 Internal Medicine, University of Missouri, Columbia, USA; 3 Internal Medicine, University of Missouri Health Care, Columbia, USA; 4 Infectious Diseases, University of Missouri, Columbia, USA; 5 Department of Medicine, Division of Pulmonary and Critical Care, University of Missouri School of Medicine, Columbia, USA

**Keywords:** salmonellosis, pneumonia, bacteremia, infectious aortitis, salmonella typhimurium

## Abstract

Nontyphoidal *Salmonellae* (NTS) often cause self-limiting gastroenteritis in adults, known as salmonellosis. Salmonellosis has remained controlled in the United States due to intensive measures. Infrequently, these patients develop bacteremia and local or disseminated infections after salmonellosis. NTS endovascular infections are frequent in patients with immunosuppression, indwelling prosthetic vascular grafts, atherosclerotic vascular disease, or aortic aneurysms. NTS endovascular infections are uncommon in immunocompetent adults. Similarly, other focal extraintestinal infections such as pneumonia are also rare. A PubMed review of the medical literature reveals few cases in healthy adults with bacteremia, pneumonia, and acute infectious thoracic aortitis with dissection due to *Salmonella typhimurium*. We present an elderly White male with salmonellosis followed by *S. typhimurium* bacteremia with pneumonia and an acute thoracic aortic dissection three weeks later. He was treated successfully with endovascular repair and antibiotics.

## Introduction

Acute infectious aortitis is an infrequent clinical entity with devastating consequences if left untreated. A mycotic aneurysm is defined as vascular aneurysmal changes due to infection. Mycotic aortic aneurysms (MAA) can involve any segment, and management is arduous with thoracic segment involvement. The incidence of MAA in the western world is 0.6-2%, whereas, in Taiwan, it is 13% [1]. Infectious causes account for a tiny fraction of aortitis, and they include *Staphylococcal* species, *Streptococcus* *pneumoniae*, *Salmonella* species, *Mycobacterium* *tuberculosis*, and *Treponema* *pallidum*. *Staphylococcal* species are the most common cause in developed countries. A frequent global cause a few decades back, *Salmonella* is now seen only in developing countries. Nontyphoidal *Salmonellae* (NTS) are responsible for foodborne infections and usually cause self-limited gastroenteritis (GE) called salmonellosis (a reportable condition) with a prevalence of 1.2 million cases in the United States [2]. In a fraction of patients with risk factors, salmonellosis causes bacteremia and extraintestinal infections. Bacteremia is often associated with vascular infections. Here, we describe an elderly White male presenting with an acute descending thoracic MAA with dissection after a recent history of salmonellosis followed by NTS bacteremia with pneumonia. Based on the PubMed medical literature review, *Salmonella typhimurium* MAA is uncommon in the western world, and pneumonia is a rare presentation seen in less than 10 patients [1,3,4].

## Case presentation

**Figure 1:**
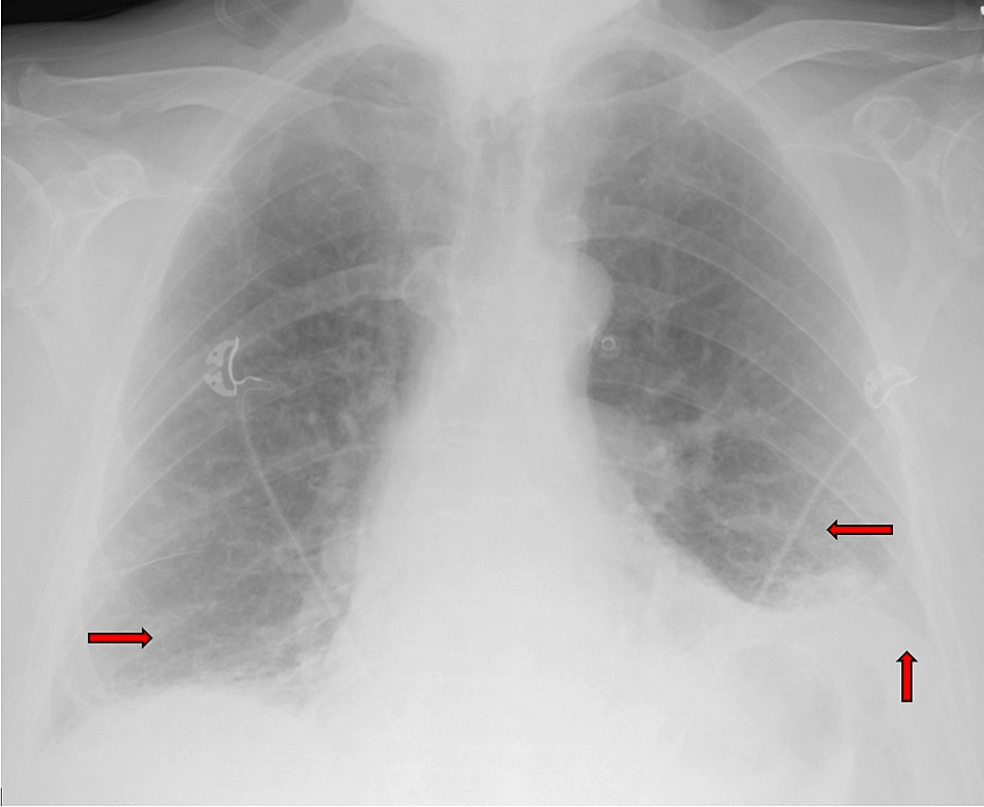
A portable chest X-ray revealed bibasilar infiltrates (horizontal red arrows) and left trace pleural effusion (vertical red arrow).

An 89-year-old male presented to an outside hospital with fever, chills, generalized fatigue, and mild diarrhea for a week, and yellowish productive cough and worsening dyspnea for four days. His past medical history was positive for chronic obstructive pulmonary disease (COPD), for which he was on home oxygen at two liters, hypertension, hyperlipidemia, and stage III chronic kidney disease. His home medications included an albuterol inhaler, benazepril, fish oil, aspirin, and tamsulosin. Personal history was positive for 80 packs a year of smoking and drinking six packs of beer daily for the last 15 years. At admission, he was afebrile with tachycardia (120 beats/minute), tachypnea (30 breaths/minute), normotensive, and hypoxic (oxygen saturation of 84% on room air and 98% on 3 liters nasal cannula). Clinical exam revealed an elderly male with minimal distress and bilateral lower lobe bronchial breath sounds. A portable chest X-ray revealed bibasilar infiltrates with left pleural effusion (Figure 1).

**Figure 2:**
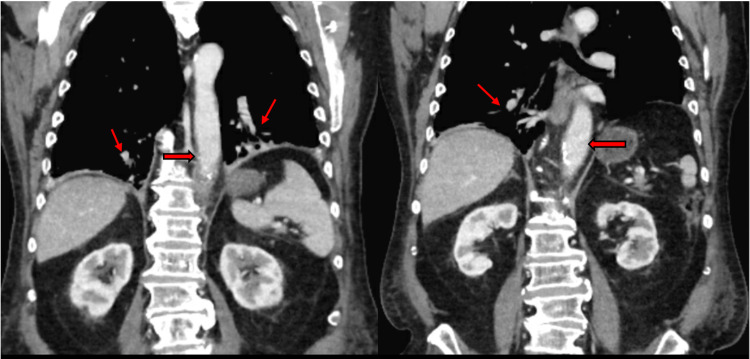
CT of the chest, abdomen, and pelvis with contrast revealed bibasilar parenchymal consolidation (oblique red arrows) and mild dilation of the proximal small bowel with no evidence of acute pulmonary embolism or aortic dissection (horizontal red arrows).

Labs were significant for leukocytosis, lactic acidosis, elevated cardiac enzymes, negative sputum culture, and positive blood culture for *Salmonella*. The transthoracic echocardiogram was unremarkable. Computed tomography (CT) scan of the chest, abdomen, and pelvis with contrast revealed bibasilar parenchymal consolidation and mild proximal small bowel dilation with no acute pulmonary embolism or aortic dissection (Figure 2).

He was diagnosed with acute sepsis due to *Salmonella* bacteremia and multilobar bacterial pneumonia, myocardial demand ischemia, and acute COPD exacerbation. He was empirically treated with intravenous (IV) antibiotics ceftriaxone (1 gm/day) and azithromycin (500 mg/day). He also received intravenous solumedrol and Duoneb for acute COPD exacerbation. The blood culture returned positive for *S. typhimurium*, which was sensitive to ampicillin, trimethoprim/sulfamethoxazole, and levofloxacin. He was discharged home on day three with a week of oral ciprofloxacin 500 mg twice a day and five days of oral prednisone (40 mg daily). He completed the prescribed course of medications.

Post antibiotic completion, he felt weak, tired, and dyspneic with cough, had chills and intermittent fever with a maximum temperature of 38.8ºC during the following week, and returned to the emergency department approximately three weeks after the initial symptoms. Clinical examination was normal with stable vital signs other than hypertension of 190/80 mmHg, necessitating a nicardipine drip treatment.

**Figure 3:**
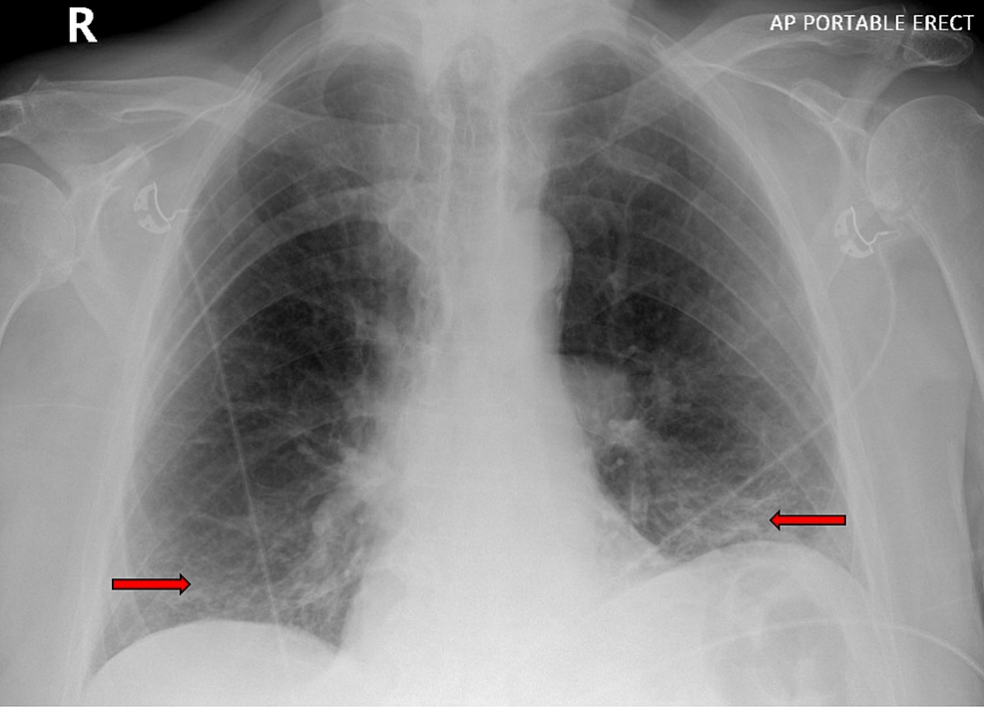
A portable chest X-ray revealed bibasilar infiltrates (horizontal red arrows) with improvement and no worsening.

Laboratory investigations were significant for an elevated creatinine and a negative test for coronavirus disease 2019 (COVID-19) (Table 1). Blood cultures obtained before antimicrobial administration revealed no growth. Chest X-ray portable revealed bibasilar infiltrates with an improvement compared to previous imaging (Figure 3).

**Table 1:** Outside hospital laboratory results. COVID-19 = coronavirus disease 2019; PCR = polymerase chain reaction.

Outside hospital laboratory results	
(1) Complete blood cell count (CBCD)	Within normal limits
(2) Complete metabolic panel (CMP)	Elevated creatinine at 1.9 mg/dL
(3) Urine analysis	Within normal limits
(4) Troponin T	Within normal limits
(5) COVID-19 nasopharyngeal PCR	Negative

**Figure 4:**
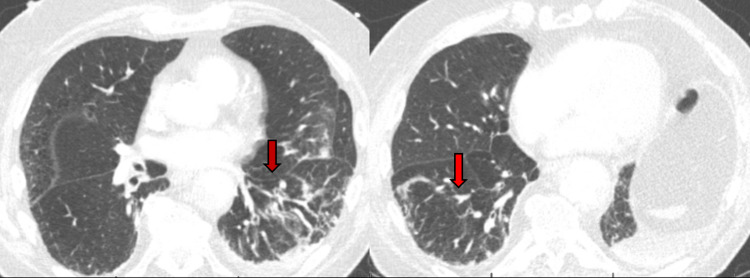
CT of the chest with contrast revealed improving multilobar pneumonia (red inverted vertical arrows).

**Figure 5:**
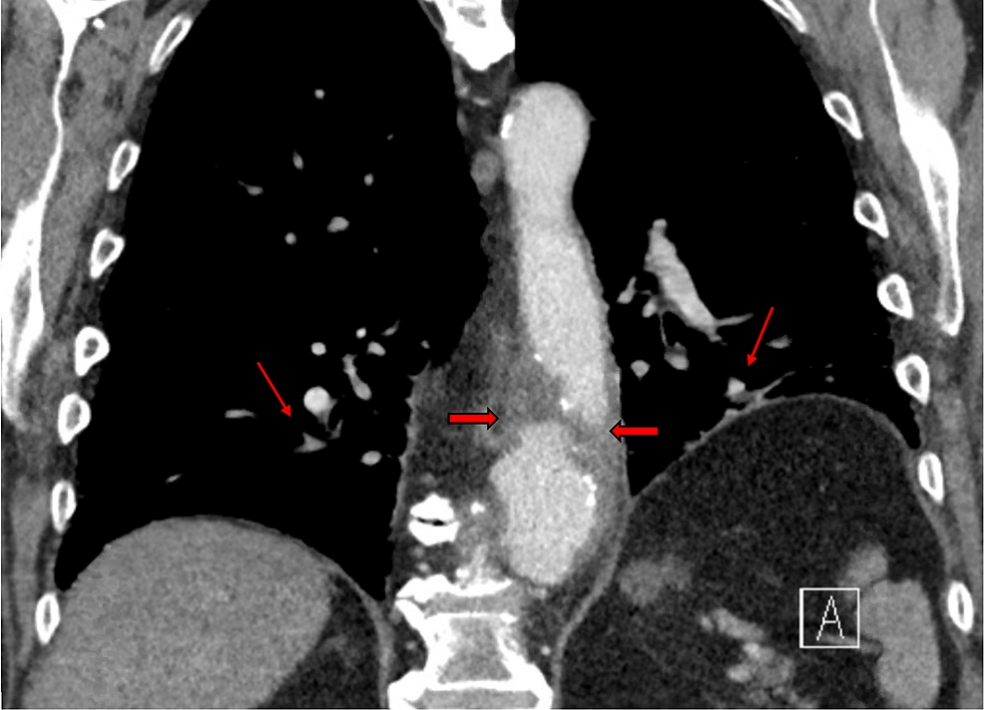
CT of the chest with contrast revealed improving multilobar pneumonia (oblique red arrows) and a new aneurysmal dilatation of the thoracic aorta with an acute dissection (horizontal red arrows). The aorta size measured 3.9 x 4.4 cm in the transverse dimension compared to 2.9 x 3 cm previously. The aorta margins were partially ill-defined and clearly at risk for rupture.

CT chest with contrast compared to the one done at the earlier admission revealed improving multilobar pneumonia (Figure 4) and a new thoracic aortic aneurysm with an acute dissection involving mid to distal descending section (Figure 5). He was started on IV fluids, levofloxacin (based on prior antimicrobial susceptibilities), antihypertensives, and transferred to our institution for acute vascular surgical intervention.

On arrival at our institution, his vital signs were stable and clinical examination was normal. He was continued on oxygen supplementation, and IV nicardipine was changed to IV esmolol for blood pressure control.

Laboratory results were significant for elevated inflammatory markers and procalcitonin (Table 2). Repeat blood cultures were negative, and the patient continued on levofloxacin. Human immunodeficiency virus and syphilis serology were negative. Transthoracic echocardiogram was normal.

**Table 2:** Laboratory results at our institution.

Laboratory results at our institution	
(1) Complete blood cell count (CBCD)	Within normal limits
(2) Complete metabolic panel (CMP)	Within normal limits
(3) International normalized ratio (INR)	1.2
(4) Troponin I	Within normal limits
(5) Procalcitonin	Elevated at 0.46 ng/mL (0.05-0.25)
(6) Erythrocyte sedimentation rate (ESR)	Elevated at 56 mm/hour
(7) C-reactive protein (CRP)	Elevated at 29.22 mg/dL

**Figure 6:**
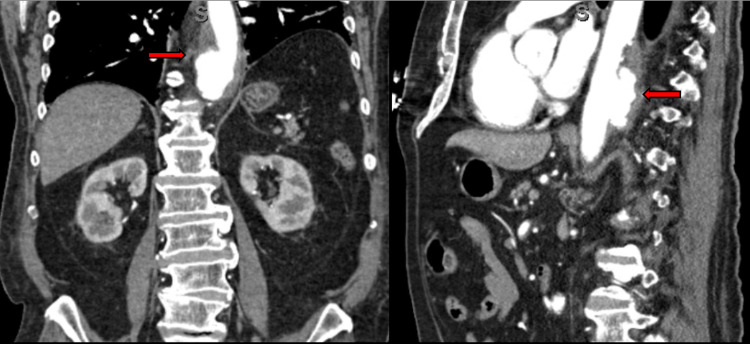
CT angiogram of the chest, abdomen, and pelvis revealed a distal descending aorta dilated aneurysm of 7.6 cm with a proximal fusiform aspect and the presence of a moderate plaque or thrombus along the posterior aspect of the aorta and displaced intimal flap (horizontal red arrows). Moderate atherosclerotic plaque with calcified and noncalcified plaque was present within the aortic arch and descending aorta.

CT angiogram of the chest, abdomen, and pelvis revealed a distal descending aortic aneurysm of 7.6 cm with a proximal fusiform aspect and a moderate plaque or thrombus along the posterior aspect of the aorta and displaced intimal flap. The aneurysm measured 3.4 x 3.6 cm. Inferiorly, there was a saccular aneurysmal dilatation seen posteriorly without a well-defined posterior wall measuring 3.7 x 4.3 cm (Figure 6). Findings were indicative of a penetrating ulcer and pseudoaneurysm with an intramural hematoma. Vascular surgery recommended acute endovascular repair as clinical history was indicative of an acute descending thoracic MAA with dissection due to *S. typhimurium* (recent *S. typhimurium* bacteremia). The infectious disease (ID) team agreed with antimicrobial therapy and surgical intervention. The patient underwent ultrasound-guided thoracic endovascular aortic repair (TEVAR). The endovascular graft was placed during an active infection, and the ID team recommended six weeks of levofloxacin after TEVAR followed by chronic suppressive antimicrobial therapy. The patient followed up at the ID clinic in six weeks and was transitioned to oral quinolones for chronic suppression.

## Discussion

NTS reservoir is multiple animals, and human infection occurs due to contaminated dairy products, undercooked ground meat, eggs, and fresh produce contaminated with animal waste [2]. Bacteremia is seen in 8% of patients with NTS GE [2]. Around 5% to 10% of these patients develop localized infections, whereas 9% to 25% develop endovascular infections, especially in patients older than 50 years [2]. Preceding diarrhea is observed in 25% of patients with aortitis [5]. NTS infections induced mortality is more frequent in the high-risk population, including the newborns, elderly, long-term care residents, and immunocompromised patients. At-risk patients include patients with diabetes mellitus, chronic liver or renal disease, sickle cell disease, malignancy, stem cell transplant, human immunodeficiency virus infection, corticosteroid, or biologic therapy [2,4]. Endovascular infection commonly involves the aorta, especially the abdominal aorta [2,4]. NTS endovascular infections are seen in patients with persistent or high-grade bacteremia. Risk factors to acquire it include atherosclerosis, aortic aneurysms, pre-existing valvular heart disease, and prosthetic vascular grafts [4]. In comparison to gram-positive organisms, *Salmonellae* are more destructive due to their invasive nature, causing aortic rupture [6]. Once infected, the bacterial growth causes diffuse suppurative or focal arteritis, disruption of the intima, and media weakening the wall with or without aneurysmal changes increasing the risk of rupture [7]. Clinical diagnosis of MAA is demanding due to nonspecific signs and symptoms and an acute to a subacute presentation with a mean symptom duration of 38 days before diagnosis [3,8]. Classic manifestations include fever, abdominal pain, pulsatile mass, and positive blood cultures [8]. Positive blood cultures are seen in 50% to 82% of patients [1,4,6]. A rupture rarely occurs without any specific signs or symptoms [8]. A transthoracic or transesophageal echocardiogram is mandatory to rule out infective endocarditis. A scoring system NTS vascular infection (NTSVI) predicts vascular infection risk in adults > 50 years with bacteremia. An NTSVI score ≥ 1 recommends obtaining a CT angiogram [9].

In suspected NTS bacteremia or endovascular infection, empirical antibiotic therapy with a third-generation cephalosporin and a fluoroquinolone should be initiated until susceptibilities are available [2]. No guidelines or randomized control trials exist to guide and determine antimicrobial duration. A recent study revealed a five-year survival rate of 71% with TEVAR comparable with survival to open MAA repair (74.9%) of the abdominal aorta [1,10]. The NTS MAA mortality rate in the 1960s was >75% and had decreased to 40% by 1984 [6]. The overall mortality from *Salmonella* aortitis is 60%, while mortality with antibacterial therapy alone is 96% and 40% with a therapeutic combination of antibiotics and surgical repair [3,11]. Finally, an ideal approach is to treat open MAA repair with antibiotics for six weeks, whereas with TEVAR, the plan is to continue the patients on chronic oral suppressive antimicrobial therapy after six weeks [2]. In our patient, the probable source of infection was poached eggs from a local farm run by his son, and the local state health department was notified. His comorbid conditions put him at risk for NTS bacteremia, which is rare [12]. Pulmonary infection with NTS is an infrequent clinical presentation seen in elderly and diabetic patients [13]. Sputum cultures have a low diagnostic yield in pneumonia and were negative in our patient (obtained after antibiotic administration) [14]. His risk factors and clinical variables increased the predictability of the blood culture in identifying the causative agent [15]. Antimicrobial therapy at the initial admission was inadequate as *Salmonella* pneumonitis is treated with antibiotics at least for two weeks as it has a high mortality of 25% to 60% [2,16]. NTSVI score was ≥2 in our patient, and CT imaging can miss the early disease. He was a poor surgical candidate and underwent successful endovascular intervention and antimicrobial therapy. A lack of vaccines makes the food handler’s hygiene improvement important and maintaining time-temperature standards critical in controlling salmonellosis [2].

## Conclusions

Blood cultures in severe community-acquired bacterial pneumonia help identify uncommon agents, and the empirical regimen and duration might not cover them. It is critical to know that NTS pneumonia antimicrobial therapy duration is longer than usual. NTSVI score can be used in the elderly with NTS bacteremia to predict the vascular infection risk. An early diagnosis and aggressive bactericidal antibiotic therapy with endovascular intervention yield the best result in high-risk patients with NTS bacteremia. Improved diagnostic imaging, timely effective antimicrobial therapy, and endovascular interventional advancements have resulted in a decline in morbidity and mortality.
